# Resting energy expenditure under fasting conditions is primarily explained by fat-free mass rather than cardiac autonomic markers

**DOI:** 10.3389/fendo.2026.1861379

**Published:** 2026-06-29

**Authors:** Andrea Geißler, Rebecca Spies, Kathrin Popp, Gisa Ufer, Roza Sabia, Marc N. Jarczok, Benedict Herhaus, Andreas Peter, Martin Heni

**Affiliations:** 1 Division of Endocrinology and Diabetology, Department of Internal Medicine I, Ulm University Hospital, Ulm, Germany; 2 Department of Psychosomatic Medicine and Psychotherapy, Ulm University Hospital, Ulm, Germany; 3 Medical Psychology and Medical Sociology, University Medical Center of the Johannes Gutenberg University Mainz, Mainz, Germany; 4 Department for Diagnostic Laboratory Medicine, Institute for Clinical Chemistry and Pathobiochemistry, Eberhard Karls University Tübingen, Tübingen, Germany; 5 German Center for Diabetes Research (DZD e.V.), Neuherberg, Germany; 6 Institute for Diabetes Research and Metabolic Diseases of the Helmholtz Center Munich at the University of Tübingen, Tübingen, Germany

**Keywords:** autonomic nervous system, catecholamines, fat-free mass, heart rate variability, indirect calorimetry, resting energy expenditure

## Abstract

**Background:**

Resting Energy Expenditure (REE) represents the largest component of total daily energy expenditure. While fat-free mass (FFM) is its primary predictor, substantial interindividual variability remains unexplained. The sympathetic nervous system has been implicated in the regulation of energy expenditure, but its contribution to REE under fasting conditions in humans is not yet determined.

**Methods:**

We investigated the relative contributions of body composition, circulating catecholamines, and cardiac autonomic modulation to REE in 38 healthy young participants following an overnight fast. REE was assessed by indirect calorimetry and FFM by bioelectrical impedance analysis. Cardiac autonomic activity was quantified through heart rate variability (HRV) analysis (time- and frequency-domain). Plasma epinephrine and norepinephrine were determined in a subsample (*n* = 19).

**Results:**

In a multivariable model including FFM, sex, and age, FFM was the dominant determinant of REE (R^2^ = 0.90, p < 0.001). Sex contributed independently, whereas age showed no significant association. Circulating epinephrine was positively associated with REE (p = 0.024), while norepinephrine was not. None of the HRV-derived parameters was significantly associated with REE.

**Conclusion:**

Under basal fasting conditions, REE is primarily associated with FFM, with an additional association with circulating epinephrine. Given the absence of associations with norepinephrine and HRV-derived parameters, the findings suggest a potential role of circulating catecholamines in interindividual variability in REE. However, direct conclusions regarding the physiological mechanisms involved cannot be drawn from the present observational study. HRV-derived cardiac autonomic markers were not associated with REE under standardized fasting conditions.

## Introduction

Because energy expenditure accounts for a large proportion of daily energy turnover and is central to long-term body weight regulation and metabolic health (1), its determinants are of considerable interest. Resting energy expenditure (REE), defined as the energy required to maintain basic physiological functions at rest and commonly assessed by indirect calorimetry, represents a major determinant of total daily energy expenditure. A relatively low REE to body composition may contribute to positive energy balance and weight gain, thereby increasing the risk of obesity and type 2 diabetes.

REE is primarily determined by fat-free mass (FFM) (2, 3), with skeletal muscle representing the largest quantitative component of FFM. However, substantial interindividual variability in REE remains unexplained by body composition alone, suggesting the involvement of additional regulatory mechanisms (3).

The sympathetic nervous system (SNS) is a major regulator of energy metabolism. Its principal neurotransmitter, norepinephrine, acts in metabolically relevant target tissues including adipose tissue, liver, and skeletal muscle, which are sympathetically innervated and express adrenergic receptors (4). Through these pathways, sympathetic activation promotes lipolysis, substrate mobilization, and thermogenesis, thereby increasing metabolic rate (5). This makes a contribution of SNS activity to interindividual differences in REE physiologically plausible. However, the extent to which SNS activity contributes to REE under fasting conditions remains unclear (6).

In humans, SNS activity can be assessed only indirectly, primarily through circulating catecholamines or by analyzing heart rate variability (HRV). HRV reflects cardiac autonomic modulation and is widely used as a noninvasive marker of autonomic function.

Specifically, HRV analysis includes parameters in the time domain, such as the root mean square of successive differences (RMSSD), which primarily reflects parasympathetic (vagal) activity. In contrast, frequency domain parameters, including high-frequency (HF) and low-frequency (LF) power, are commonly used to characterize parasympathetic activity and mixed autonomic modulation, sometimes summarized using the LF/HF ratio (7). However, while these markers provide insight into cardiac autonomic control, they may not adequately capture sympathetic activity in metabolic tissues beyond the heart (8). Consequently, the extent to which circulating catecholamines or HRV-derived parameters provide information on REE beyond body composition under fasting conditions remains unclear.

Therefore, the present study aimed to investigate the relative contributions of FFM, catecholamines, and distinct HRV indices (spanning both time and frequency domains) parameters to REE under standardized fasting conditions in healthy adults.

## Materials and methods

This observational cross-sectional study included 38 healthy volunteers, who were healthy without any known medical condition or regular medication. Female participants included both hormonal contraceptive users and non-users. All participants provided written informed consent and the local Ethics Committee approved the protocols (300/2022 and 81/2024). All assessments were performed under standardized basal conditions in the early morning following an overnight fast of at least 10 hours. Participants remained in a supine position in a thermoneutral environment throughout the measurement period.

### Indirect calorimetry

REE and respiratory quotient were determined by indirect calorimetry using a ventilated canopy system (COSMED, Rome, Italy). Following a 2-minute acclimatization period, gas exchange was continuously recorded over 10 minutes. The system was calibrated prior to each measurement using standardized reference gases. Oxygen consumption (VO_2_) and carbon dioxide production (VCO_2_) were used to calculate energy expenditure according to the abbreviated Weir equation (9): REE (kcal/day) = [3.941 x VO_2_ (L/min) + 1.106 x VCO_2_ (L/min)] x 1440.

Respiratory quotient was used as an index of substrate utilization, with values between 0.7 and 0.8 considered indicative of fasting conditions.

A sensitivity analysis compared measured REE with estimates derived from the Harris-Benedict equation (10).

### Assessment of body composition through bioelectrical impedance analysis

Body composition was assessed using multi-frequency bioelectrical impedance analysis (BIA) (BIA 101 BIVA PRO; Akern, Florence, Italy) operating at 50 kHz. Data were processed using the Bodygram HBO software (Akern) to estimate FFM. The method measures resistance (R) and reactance (X_c_) of body tissues to an applied alternating current (11). Based on impedance measurements, total body water was estimated and partitioned into intracellular water and extracellular water. FFM was derived from total body water using device-specific prediction equations implemented in the manufacturer’s software. As an indirect method, BIA provides only a composite estimate of FFM and does not allow separation of individual tissue compartments.

### Electrocardiography and HRV-analysis

Cardiac autonomic activity was assessed using a short-term 5-minute measurement obtained while participants were in the supine position. The high-resolution electrocardiography (sample rate: 1000 samples/sec) was recorded with the BIOPAC MP150 system (BIOPAC Systems Inc., Goleta, CA, USA). RR intervals were exported and analyzed using Kubios HRV Premium software (Version 3.5, Kuopio, Finland) (12).

Specifically, the RMSSD was calculated as a time-domain parameter representing parasympathetic activity. Frequency-domain analysis was performed using the Fast Fourier Transform to determine LF (0.04-0.15 Hz) and HF (0.15-0.40 Hz) power, as well as the LF/HF ratio, as an index of cardiac sympathovagal modulation (7). Data quality was ensured through automated beat correction (13) and the smoothness priors method of trend component rejection (14). Artifact rates remained below 3% in all recordings, meeting established methodological standards for reliable HRV analysis (7).

### Catecholamine measurement

Plasma catecholamines were assessed in a subsample of *n* = 19. Blood samples were collected in EDTA tubes, placed on ice, centrifuged within 30 minutes, and stored at -80 °C until analysis by high-performance liquid chromatography with electrochemical detection.

### Statistical analysis

All analyses were conducted using R (Version 2026.01.1 + 403). Continuous variables are presented as median [interquartile range (IQR)] and 95% confidence intervals. Standard errors of the estimates (SEE) are presented for as a measure of accuracy in regression analyses. Data distribution was assessed using the Shapiro-Wilk test. HRV-derived autonomic parameters were ln-transformed to achieve normality prior to correlation analyses.

Associations between REE and its potential determinants were evaluated using both Pearson’s correlation coefficient (r) for transformed variables and Kendall’s τ for non-parametric analyses.

Independent associations of FFM, catecholamines, and HRV on REE were evaluated using multivariable linear regression models adjusted for age and sex. Model assumptions were evaluated by inspection of residual plots and assessment of normality and homoscedasticity. Multicollinearity between predictors was examined using variance inflation factors (VIF), with all values indicating no relevant collinearity (VIF < 5).

The selection of variables included in the regression models was based on established physiological relevance and prior literature. Statistical significance was defined as p < 0.05.

A *post-hoc* power analysis based on the observed effect size of the correlation between epinephrine and REE revealed an achieved power (1-beta error probability) of 0.95, indicating the sample size was sufficient to statistically addressed this question.

## Results

A total of 38 healthy volunteers were included (16 males and 22 females; age 22.9 ± 2.6 years; BMI 21.7 ± 1.8 kg/m^2^). Anthropometric, metabolic, and HRV characteristics are summarized in **Tables 1** and **2**. All participants completed the study protocol as planned, and no adverse events were reported during the assessment period.

**Table 1:** Anthropometric and metabolic characteristics of the study population (*n* = 38).

Characteristics	Men (n = 16)	Women (n = 22)
Age (years)	23.0 [21.8-25.2]; (95% CI: 22.2-24.7)	22.5 [22.0-23.8]; (95% CI: 21.6-23.7)
Height (cm)	181.0 [176.5-185.0]; (95% CI: 179.0-184.0)	162.5 [160.8-167.2]; (95% CI: 162.6-164.9)
Weight (kg)	73.5 [69.0-81.1]; (95% CI: 71.9-77.1)	58.2 [54.9-59.9]; (95% CI: 55.0-58.1)
BMI (kg/m^2^)	22.9 [21.3-23.8]; (95% CI: 22.2-22.9)	21.5 [20.5-22.5]; (95% CI: 20.5-21.8)
Cortisol (nmol/l)	637.0 [558.5-653.0]; (95% CI: 501.0-663.7)	497.0 [404.5-647.0]; (95% CI: 432.4-600.6)
FFM (kg)	59.6 [56.2-64.5]; (95% CI: 57.8-63.4)	43.2 [41.8-44.6]; (95% CI: 41.2-44.1)
HbA1c (mmol/mol)	31.0 [29.5-33.5]; (95% CI: 29.7-33.0)	32.5 [29.8-33.2]; (95% CI: 30.2-33.7)
Resting heart rate (bpm)	63.2 [59.1-70.8]; (95% CI: 60.3-66.7)	69.1 [64.9-75.7]; (95% CI: 57.9-73.7)
REE (kcal/day)	1821 [1739-1948]; (95% CI: 1763-1894)	1396 [1359-1431]; (95% CI: 1367-1407)
TSH (mU/l)	2.9 [1.6-3.3]; (95% CI: 1.8-3.3)	2.2 [1.4-3.0]; (95% CI: 1.8-3.5)
Plasma epinephrine (pg/ml)^a^	6.40 [3.00-9.10]; (95% CI: 4.76-9.49)	3.00 [3.00-4.90]; (95% CI: 3.31-4.95)
Plasma norepinephrine (pg/ml)^a^	57.30 [54.20-65.20]; (95% CI: 52.71-66.37)	55.55 [42.10-74.68]; (95% CI: 48.31-76.23)

Data are presented as median [IQR]; (95% CI). ^a^Catecholamines were only available in a sub-sample (n = 19); values below the limit of detection were set to 3.0 pg/ml. Abbreviations: BMI, body mass index; CI, confidence interval; FFM, fat-free mass; HbA1c, glycated hemoglobin; bpm, beats per minute; IQR, interquartile range; REE, resting energy expenditure; TSH, thyroid-stimulating hormone; SD, standard deviation.

**Table 2:** Heart rate variability measures of the study population.

HRV measures	Men (n = 16)	Women (n = 22)
SDNN (ms)	108.5 [103.2-133.6]; (95% CI: 95.4-137.8)	110.1 [80.3-134.7]; (95% CI: 80.5-136.5)
RMSSD (ms)	69.0 [48.7-83.8]; (95% CI: (55.2-91.6)	50.4 [37.3-103.7]; (95% CI: 44.2-120.6)
LF power (ms2)	1217 [797-2839]; (95% CI: 996-4075)	1142 [597-2717]; (95% CI: 902-4727)
HF power (ms2)	1237 [947-2294]; (95% CI: 953-2875)	968 [283-1813]; (95% CI: 626-3952)
LF/HF ratio	1.25 [0.78-3.15]; (95% CI: 0.95-2.59)	1.51 [0.76-2.57]; (95% CI: 1.36-2.46)

Data are presented as median [IQR]; (95% CI). Abbreviations: CI, confidence interval; HF, high frequency; HRV, heart rate variability; IQR, interquartile range; LF, low frequency; RMSSD, root mean square of successive differences; SDNN, standard deviation of NN intervals.

### Determinants of REE

**Figure 1:**
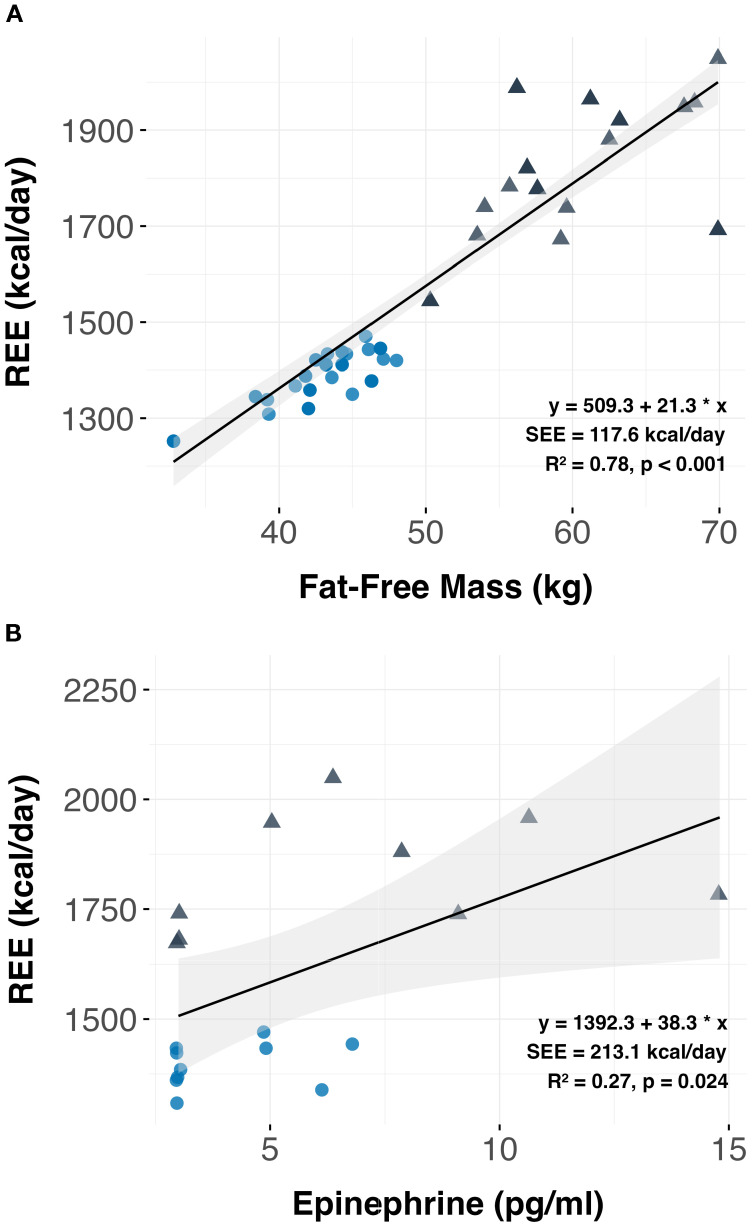
Associations of fat-free mass and epinephrine with resting energy expenditure. **(A)** Scatter plot of fat-free mass (kg) and resting energy expenditure (kcal/day) (n = 38). **(B)** Scatter plot of epinephrine (pg/ml) and resting energy expenditure (kcal/day) (n = 19). Black lines represent the pooled linear regression across all subjects. The shaded grey areas indicate the 95% confidence interval of the regression line. Data points are color-coded by sex (blue circles: female, dark grey triangles: male). REE, resting energy expenditure; SEE, standard error of estimate.

A multivariate model including FFM, sex, and age explained 90.3% of the variance in REE (p < 0.001, SEE = 77.7 kcal/day). The univariate association between FFM and REE explained 85% of the variance (SEE = 92.7 kcal/day; **Figure 1A**). FFM was the primary determinant of REE (estimate = 13.53 kcal/kg FFM/day, p < 0.001). Males had higher REE (estimate = 204.1 kcal/day, p < 0.001), whereas age showed no significant association (p = 0.14). The relationship between REE and FFM was not significantly different between sexes (p for interaction = 0.79).

To assess the potential influence of the REE estimation method, a sensitivity analysis was conducted using the weight-based Harris-Benedict equation. REE values derived from both approaches showed a correlation of r = 0.999 (p < 0.001). In a multivariate model with the Harris-Benedict-derived REE as the dependent variable, FFM was associated with REE (p < 0.001), and the model explained 81.6% of the variance.

### Sympathetic activity and autonomic control

Univariate analysis demonstrated a positive association between epinephrine and REE (SEE = 213.1 kcal/day; R^2^ = 0.27, p = 0.024; **Figure 1B**). This association remained significant after adjustment for FFM, sex, and age (p = 0.016). Norepinephrine was not significantly associated with REE (p = 0.62).

However, none of the tested HRV-derived parameters (including RMSSD, SDNN, LF and HF power) showed a significant association with REE (all p ≥ 0.18, **Table 3**).

**Table 3:** Correlation of REE with anthropometric and physiological parameters.

Parameter	Correlates of REE	Kendall (τ)^[2]^
	Pearson (r)^[1]^	
Anthropometrics		
FFM	0.93***	0.78**
Endocrine Markers		
Epinephrine	0.52*	0.41*
Autonomic Cardiac Indices		
Mean Heart Rate	-0.08	0.14
LF power	-0.04	0.08
HF power	-0.04	0.05
RMSSD	0.01	0.02

n = 38. REE and FFM, and mean heart rate were analyzed without transformation; endocrine and autonomic frequency-domain parameters were ln-transformed prior to analysis to satisfy the assumption of normality. ^a^Catecholamines were only available in a sub-sample (n = 19). ^[1]^Pearson correlation on ln-transformed data. ^[2]^Kendall’s (τ) on raw data. Abbreviations: FFM, fat-free mass; HF, high frequency; LF, low frequency; RMSSD, root mean square of successive differences. *p<0.05, **p<0.01, ***p<0.001.

## Discussion

In the present study, we investigated potential determinants of REE under standardized fasting conditions. We confirm that FFM is a strong predictor of REE, consistent with its established association with basal metabolic rate (3). In addition, circulating epinephrine was associated with REE. Given the absence of associations with norepinephrine and HRV-derived parameters, circulating catecholamines may represent a factor associated with variability in REE beyond body composition. However, given the observational design, causality cannot be inferred, and this finding should be interpreted as exploratory.

Beyond these methodological constraints, the present findings may be relevant for human energy homeostasis and obesity research. The association between circulating epinephrine and REE is consistent with previous findings on catecholamine signaling and energy expenditure (15, 16). This is consistent with evidence that interindividual differences in catecholamine signaling contribute to variability in energy expenditure not explained by FFM alone. Prior work has linked catecholamine signaling and sympathoadrenal activity to interindividual variability in energy expenditure and obesity susceptibility (15, 17). Our findings are consistent with previous observations linking catecholamine signaling to variability in REE. However, the present data do not allow conclusions regarding the specific physiological pathways underlying this association.

These observations may be relevant for future research on endocrine, non-structural determinants of metabolism and may inform more refined metabolic phenotyping approaches in obesity research.

The association of REE with circulating epinephrine, but not norepinephrine, may reflect differences in how these biomarkers relate to resting metabolism. While epinephrine acts as a global metabolic signal, plasma norepinephrine is often considered a limited proxy for overall sympathetic activity due to its organ-specific spillover patterns (18). Nevertheless, these interpretations remain tentative, particularly given the single time-point assessment of catecholamines and the limited sample size in the subsample analysis.

No association was observed between HRV-derived parameters and REE, consistent with HRV reflecting cardiac autonomic modulation rather than whole-body sympathetic metabolic drive. Accordingly, HRV-derived measures may not fully capture autonomic influences on tissues relevant to REE. More specifically, HRV primarily captures cardiac parasympathetic and sympathetic balance and may not adequately represent organ-specific sympathetic outflow or endocrine-driven metabolic regulation (4). Consequently, the absence of HRV associations should not be interpreted as evidence against a role of autonomic mechanisms in REE regulation.

Within body composition, skeletal muscle may partially contribute to the association between fat-free mass (FFM) and resting energy expenditure (REE) due to its substantial proportion of total FFM (19, 23) and its significant role in overall energy turnover (19). However, skeletal muscle is characterized by a relatively low specific metabolic rate (approximately 13 kcal/kg/day) compared to highly metabolically active organs such as the liver, brain, heart, and kidneys (20). Furthermore, FFM captures both skeletal muscle and these highly metabolically active organs, and the relative contribution of individual tissues could not be quantified in the present study. Consequently, interindividual differences in the relative mass of high-metabolic-rate organs within FFM may contribute to residual variability in REE that is not captured by total FFM alone.

Of note, the strong relationship between DXA-derived FFM and REE is not only dependent on sex, but also on age and physical activity level (21). There is a high agreement between BIA- and DXA-derived FFM estimates for metabolic rate assessment in young, physically active populations (22), supporting the validity of the present BIA-based approach.

To address potential methodological bias with the calorimetry-based assessment of REE, a sensitivity analysis using weight-based REE equations was performed. The observed associations remained consistent across approaches, suggesting that the findings were not dependent on the specific REE assessment method applied.

Limitations include the focus on basal, fasting conditions, which may underestimate sympathetic contributions under postprandial or other stimulated states. Furthermore, HRV primarily measures cardiac activity and may not capture organ-specific sympathetic modulation. Additionally, catecholamines were assessed at a single time point in a smaller subsample, which limits statistical power and increases the risk of a Type II error. Consequently, the observed association for epinephrine should be interpreted with caution as a preliminary and considered exploratory. Finally, body composition was assessed using BIA rather than imaging-based reference methods such as DXA or MRI. BIA provides an indirect, model-based estimate of FFM derived from body water distribution (11) and does not permit differentiation between individual tissue compartments, limiting mechanistic interpretation at the tissue level. In addition, the present sample consisted exclusively of a Caucasian population, which may limit the generalizability of the findings to populations with different body composition characteristics or organ-to-muscle distributions.

In conclusion, interindividual variability in REE appears to be primarily related to body composition, particularly FFM, with a possible additional association of circulating epinephrine independent of body composition. No associations were observed for norepinephrine or HRV-derived parameters, suggesting limited relevance of these markers for interindividual variability in REE under standardized fasting conditions. Although causal pathways and tissue-specific contributions cannot be established in the present observational study, skeletal muscle may represent one physiologically relevant component underlying the association between FFM and REE. Future studies integrating imaging-based body composition assessment with direct measures of autonomic and endocrine activity are warranted to further define the physiological determinants of basal energy expenditure.

## Data Availability

The data generated during the current study are not publicly available because of privacy and ethical restrictions. Requests to access the datasets should be directed to Martin Heni martin.heni@uniklinik-ulm.de.
